# A Novel Eco-Friendly and Highly Sensitive Solid Lead–Tin Microelectrode for Trace U(VI) Determination in Natural Water Samples

**DOI:** 10.3390/s23052552

**Published:** 2023-02-24

**Authors:** Iwona Gęca, Mieczyslaw Korolczuk

**Affiliations:** Institute of Chemical Sciences, Faculty of Chemistry, Maria Curie Sklodowska University, 20-031 Lublin, Poland

**Keywords:** uranium, stripping analysis, determination, solid lead–tin microelectrode

## Abstract

For the first time a solid state lead–tin microelectrode (diameter ϕ 25 µm) was utilized for U(VI) ion determination by adsorptive stripping voltammetry. The described sensor is characterized by high durability, reusability and eco-friendly features, as the need for using lead and tin ions for metal film preplating has been eliminated, and consequently, the amount of toxic waste has been limited. The advantages of the developed procedure resulted also from the utilization of a microelectrode as a working electrode, because a restricted amount of metals is needed for its construction. Moreover, field analysis is possible to perform thanks to the fact that measurements can be carried out from unmixed solutions. The analytical procedure was optimized. The proposed procedure is characterized by two orders of magnitude linear dynamic range of U(VI) determination from 1 × 10^−9^ to 1 × 10^−7^ mol L^−1^ (120 s of accumulation). The detection limit was calculated to be 3.9 × 10^−10^ mol L^−1^ (accumulation time of 120 s). RSD% calculated from seven subsequent U(VI) determinations at a concentration of 2 × 10^−8^ mol L^−1^ was 3.5%. The correctness of the analytical procedure was confirmed by analyzing a natural certified reference material.

## 1. Introduction

Microelectrodes are electrochemical sensors that are gaining increased importance and interest among scientists in over four decades in the field of electrochemistry, biotechnology, environmental science and medicine [1,2]. The definition of “microelectrode” is related to its size. Briefly, if one characteristic size of a particular electrode is comparable to or smaller than the size of the diffusion layer within the scope of a given experiment, then a steady state (pseudo-steady state in the course of cylindrical electrodes) is observed. Under such conditions, the electrode exhibits the properties of a microelectrode [3]. In fact, the most general and commonly used description of microelectrodes refers to electrodes that are characterized by a minimum one dimension smaller than 25 µm (e.g., a diameter) [4]. A variety of microelectrodes geometries are reported in the literature, as are their fabrication techniques and the materials from which they are produced [5,6,7,8,9,10,11].

In comparison to conventionally sized electrodes, utilization of microelectrodes results in obtaining many characteristic benefits precisely described in the literature, including an enhanced signal-to-noise ratio, a high mass transport and an immunity to ohmic drop. A very slight surface area of microelectrodes leads to obtaining a small double-layer capacitance, so the charging current is drastically reduced. Thanks to this fact, an increase in the sensitivity of determinations is observed [12,13].

Because of the very small size of microelectrodes, a miniaturization of electrochemical systems is possible, and consequently, measurements can be provided from very small volumes of the samples. Furthermore, because of a slight ohmic drop, voltammetric measurements can be conducted from diluted solutions [14,15]. As a result of the abovementioned facts, the utilization of microelectrodes reduces both the amount of toxic waste and the cost of analysis. A very important advantage of using microelectrodes is the possibility of providing measurements from an organic solvent, in contrast to macroelectrodes [16].

A spherical diffusion occurring near the microelectrodes’ surface facilitates a natural convection, and consequently, a preconcentration step during voltammetric measurements can be provided without stirring the solution [16,17,18]. As a result, field analysis and real-time monitoring are possible by means of stripping voltammetry.

The utilization of microelectrodes faces also some restrictions mainly due to their small sizes. First, in contrast to conventionally sized electrodes, the recorded currents are relatively low and, consequently, sensitive to disturbances. Moreover, the micrometer dimensions of these sensors make their construction relatively difficult.

Uranium, a heavy metal from the actinides group in the periodic table of elements, has great interest because of both its important use in the nuclear industry as an energy source and its radioactive, hazardous, toxicological and accumulative properties [19]. Uranium, existing in two stable oxidation states, +IV and +VI, is a naturally occurring and ubiquitous element found in various environmental samples such as soil, rocks, seas, oceans and microorganisms. Typical concentrations of uranium in soil, air, surface water and groundwater have been reported previously [20]. According to the EPA (Environmental Protection Agency), a permissible concentration level of uranium in drinking water is equal to 30 µg L^−1^ [21]. Consumption over a long period of time of drinking water with too high concentration of uranium can lead to health problems, including kidney and bone damage, cancer, an increased rate of birth defects and cardiovascular diseases [22,23,24,25,26]. The abovementioned facts lead to the conclusion that monitoring uranium content in environmental samples is a very important, urgent and interesting issue.

For determination of uranium content in the environmental samples, various analytical methods have been used involving neutron activation analysis [27,28], spectrophotometry [29,30], colorimetry [31], fluorometry [32,33], flame atomic absorption spectrometry (FAAS), electrothermal atomic absorption spectrometry (ETAAS), inductively coupled plasma optical emission spectrometry (ICP-OES), inductively coupled plasma mass spectrometry (ICP-MS) [34,35,36], X-ray fluorescence [37] and many others [38,39,40,41,42,43,44,45]. Stripping voltammetry is a very important electrochemical technique that is commonly used in environmental analysis thanks to the possibility of its on-site application. This method has been often used successfully for uranium determination by means of many different working electrodes. Despite the benefits, there are only a few literature reports concerning uranium determination using microelectrodes, including a vibrating gold microelectrode [46], a powder microelectrode [47], an SPE microelectrode [48] and a lead film microelectrode after an initial U(VI)–cupferron preconcentration on a lead film electrode with a large surface area [49]. Monitoring of uranium content in the environmental samples by means of microelectrodes seems to be an interesting topic for further research.

Recently we reported solid single lead and bismuth microelectrodes for the determination of inorganic and organic species [50,51,52]. The abovementioned sensors can be an alternative for both metal film electrodes and solid metal electrodes of traditional dimensions [53] because of (i) eliminating the need of metal ions additions to the supporting electrolyte for a metal film plating and (ii) a limited amount of metal needed for their construction.

The undoubted advantage of this type of microelectrodes is the simplicity oftheir preparation for the measurements, involving a short mechanical polishing of their surface with sandpaper and a few-seconds activation step during the measurement procedure. This fact simplifies the course of the experiment, shortens its time and makes the analytical procedures cheaper, in contrast to metal film/chemically modified/surface modified microelectrodes [54,55,56,57,58,59]. The abovementioned facts combined with the general advantages resulting from using microelectrodes make the proposed solid metal microelectrodes important, convenient and attractive electrochemical tools.

Further studies concerning construction and application of a solid metal microelectrode are presented in this paper. The paper reports for the first time utilization of a new durable, reusable and eco-friendly voltammetric sensor: a lead–tin microelectrode for uranium(VI) determination by adsorptive stripping voltammetry (AdSV). Due to the composition of the microelectrode material, the sensor proposed in the present paper is even more environmentally friendly than a previously described solid lead microelectrode [50]. The electrochemical features of the solid lead–tin microelectrode were described. The favorable experimental conditions of U(VI) determination on the solid lead–tin microelectrode were studied. The satisfying repeatability of measurements expressed with a relative standard deviation RSD 3.5 % (n = 7) was ensured by applying a short activation step at −2.5 V within 3 s before each measurement. The correctness of the developed voltammetric procedure was proved by satisfactory results of an analysis of the certified natural water reference material.

## 2. Materials and Methods

### 2.1. Apparatus

The experiments were conducted with a µAutolab analyzer made by Eco Chemie (Utrecht, The Netherlands). The measurements were provided in a 3-electrode electrochemical cell (volume of 10 mL) consisting of a solid lead–tin microelectrode as a working electrode and a platinum wire and Ag/AgCl/NaCl as auxiliary and reference electrodes, respectively. A surface of working lead–tin microelectrode was prepared mechanically every day by polishing it with an abrasive paper made by Starcke (Melle, Germany) of 2000 grit, and then a microelectrode was sonicated in deionized water for half a minute by means of an ultrasonic cleaner Sonic-3 purchased from Polsonic, Poland.

### 2.2. Reagents

TraceSELECT reagents acquired from Sigma Aldrich were taken to prepare 1 mol L^−1^ acetate buffers CH_3_COOH/CH_3_COONa. U(VI) standard solution at a concentration of 2 × 10^−2^ mol L^−1^ was obtained by dissolving (CH_3_COO)_2_UO_2_ × 2H_2_O in 0.1 mol L^−1^ HNO_3_. U(VI) working solutions at a concentration of 1 × 10^−5^ mol L^−1^ were prepared every day with an appropriate dilution of U(VI) standard solution in deionized water. A cupferron solution (N-nitrosophenylhydroxylamine ammonium salt) at a concentration of 1 × 10^−2^ mol L^−1^ was prepared every week by dissolving an appropriate amount of a reagent purchased from Merck (Darmstadt, Germany) in deionized water. Certified reference material TM 25.5 (Lake Ontario water) used for validation of the developed procedure was purchased from Environment and Climate Change (Canada). Water purified in a Millipore system was used to prepare all solutions. Lead–tin solder alloy was obtained from Alfa Aesar (Kandel, Germany).

### 2.3. Design of a Solid Lead–Tin Microelectrode

**Figure 1:**
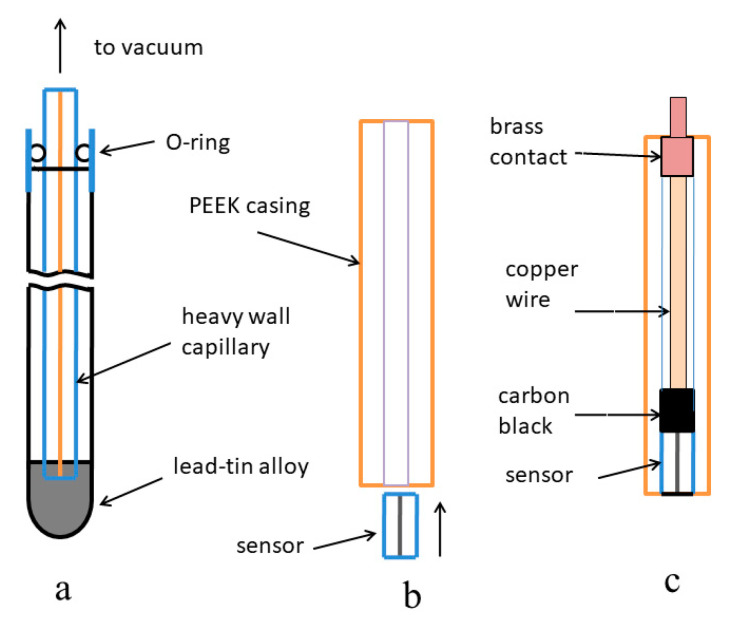
A scheme of a fabrication of a solid lead–tin microelectrode: (**a**) a setup for filling the heavy wall capillary with a lead–tin alloy; (**b**) placing a sensor in PEEK casing; (**c**) a final scheme of a construction of a solid lead–tin microelectrode.

A solid state lead–tin microelectrode (ϕ 25 µm) used as a working electrode in the described research was constructed in the following manner. First, a heavy-wall glass capillary with an outer diameter of 3 mm and an inner diameter of 25 µm was filled with a melted lead–tin solder alloy with the following composition: Pb:Sn 50:50 wt %. In order to fill the capillary with lead–tin alloy, the solder was placed in a glass tube with an inner diameter of 5 mm and a length of about 15 cm. Next, a 20–30 cm long heavy-wall capillary was placed above the alloy, as shown in Figure 1a. The tube furnace was heated to a temperature of 400 °C. A glass tube containing an alloy and a capillary was placed in a tube furnace in such a way that about 5 cm of the tube was outside the furnace. In about 10 min the lead–tin alloy was melted, and then a vacuum was connected to a heavy-wall capillary. Under these conditions, the alloy was forced into the capillary. The capillary was filled only in its heated part; sensors with a length of about 7–12 cm were usually obtained. In order to prevent the lead from evaporating into the laboratory atmosphere, a glass tube was connected with a heavy-wall capillary outside the heating part and above the tube being heated. The capillary filled with the lead–tin alloy was cut to about 7 mm long sensors, and both ends of the sensors were polished. Such a prepared sensor was placed in PEEK casing preheated to a temperature of about 200 °C, as presented in Figure 1b. An electrical contact from lead–tin microelectrode was made with the use of carbon black powder and a copper wire with a diameter of 2.5 mm. The copper wire was stabilized by screwing down the M 4 final brass contact. This method of microelectrodes construction ensures that high-stability sensors with prolonged applicationare obtained, which was confirmed (i) by using lead–tin microelectrodes for more than 5 months without significant changes in the obtained results and (ii) by features of other solid metal microelectrodes prepared in this way [50,51,52]. Another advantage of the discussed metal alloy microelectrode as compared to a solid lead microelectrode is the fact that the latter one requires intermittent polishing using an abrasive paper of 1200 grit before a final polishing with a sandpaper of 2000 grit. Intermittent polishing can be omitted when working with a lead–tin microelectrode, which proves to have high stability for this sensor probably connected with slower oxidation of its surface as compared to a solid lead microelectrode. Further reduction of lead content in the electrode material is the next significant advantage of the proposed working electrode.

### 2.4. Real Water Sample Preparation

A sample of the lake water certified reference material TM-25.5 was mineralized by UV-irradiation within 3 h. Due to the fact that the certified reference material was acidified for its stabilization, the volume of 55.8 µL 1 mol L^−1^ sodium hydroxide was added per 885 µL of mineralized TM 25.5 for pH neutralizing before measurements were performed. U(VI) concentration was determined by means of the standard measurements procedure described below.

### 2.5. Measurements Procedure

A given sample volume was added to the voltammetric cell, and then 1 mL of 1 mol L^−1^ acetate buffer CH_3_COOH/CH_3_COONa (pH 4.00) and 40 µL 1 × 10^−2^ mol L^−1^ of cupferron as a complexing agent were added. Then deionized water was added to adjust a sample to the volume of 10 mL. The measurement was conducted with a potential sequence as follows. First, the potential of −2.5 V within 3 s was applied to the working microelectrode. Second, accumulation of U(VI)–cupferron species on the surface of a microelectrode was carried out at −0.7 V for 120 s. Finally, after 10 s equilibration step square wave voltammograms were recorded, while a changing value of the potential from −0.7 to −1.1 V was applied. Frequency, amplitude and step potential were 200 Hz, 50 mV and 4 mV, respectively. The analyzed solutions were not deoxidized.

## 3. Results and Discussion

Initial experiments conducted with a solid lead–tin microelectrode showed that such a working microelectrode can be utilized for uranium(VI) ion determination with a satisfactory sensitivity and a high reproducibility by means of square wave adsorptive stripping voltammetry.

Preliminary results indicated that the proposed microelectrode can be used for providing measurements from mixed and unmixed solutions as presented in Figure S1 (in Supplementary Material), which confirms its microelectrode features. The high mass transport is a consequence of the presence of radial diffusion near the microelectrode’s surface and makes forced convection (mixing) unnecessary during the stage of accumulation without significant impact on the sensitivity of determinations (Figure S1). This is an important advantage that distinguishes microelectrodes from conventionally sized working electrodes.

This paper reports on the development of an analytical procedure of U(VI) species quantification by means of a solid lead–tin microelectrode. The obtained results are presented below.

### 3.1. Operational Potential Range of Solid Lead–Tin Microelectrode

**Figure 2:**
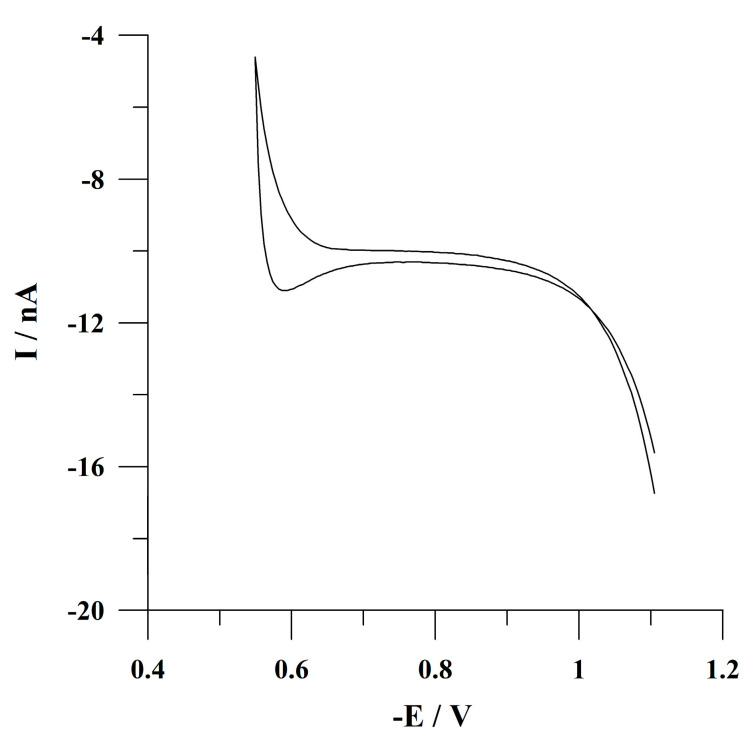
Cyclic voltammogram recorded using a solid lead–tin microelectrode from a solution containing 0.1 mol L^−1^ acetate buffer (pH 4.0). Start potential, −1.1 V; first vertex potential, −0.55 V; second vertex potential, −1.1 V. Scan rate 50 mV s^−1^.

The working potential range of the described solid lead–tin microelectrode was assessed by means of cyclic voltammetry. The measurements were performed from 0.1 mol L^−1^ CH_3_COOH/CH_3_COONa (pH of 4.00). Scan rate was equal to 50 mV s^−1^. The obtained result is presented in Figure 2. On the basis of the cyclic voltammogram shown in Figure 2, it can be concluded that the operational potential range of the proposed solid alloy microelectrode is within the range −0.6 to −1.1 V and is restricted by oxidation of microelectrode material and hydrogen ion reduction. The presented working potential window of the described microelectrode is narrower as compared to the previously reported solid lead microelectrode [50].

### 3.2. Impact of pH on U(VI)–Cupferron Signal

**Figure 3:**
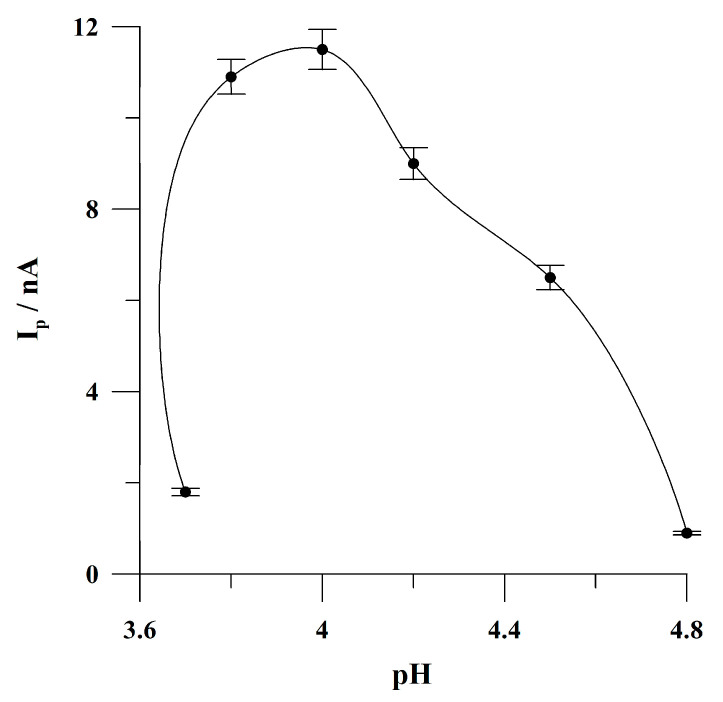
Effect of pH of acetate buffer on uranium signal. U(VI) concentration: 5 × 10^−8^ mol L^−1^. Accumulation conditions: −0.7 V, 120 s. The error bars refer to the standard deviation (n = 3).

Sample pH influences the formation of complexes of metals ions with cupferron and their subsequent adsorption on the surface of a working electrode and, consequently, the sensitivity of determinations. Thus, the impact of pH of acetate buffer on the height of U(VI) signal was investigated in a range from 3.7 to 4.8. U(VI) ion concentration was 5 × 10^−8^ mol L^−1^. Accumulation conditions were −0.7 V, 120 s. The results obtained during these studies are shown in Figure 3. It was found that the highest U(VI) signal was observed at a pH of 4.00, and this observation allows to conclude that such a condition is the most optimal for U(VI)–cupferron complex formation and its adsorption on a surface of the solid lead–tin microelectrode. Therefore, a pH of 4.00 was selected as the optimum value for further research.

### 3.3. Effect of Cupferron Concentration

**Figure 4:**
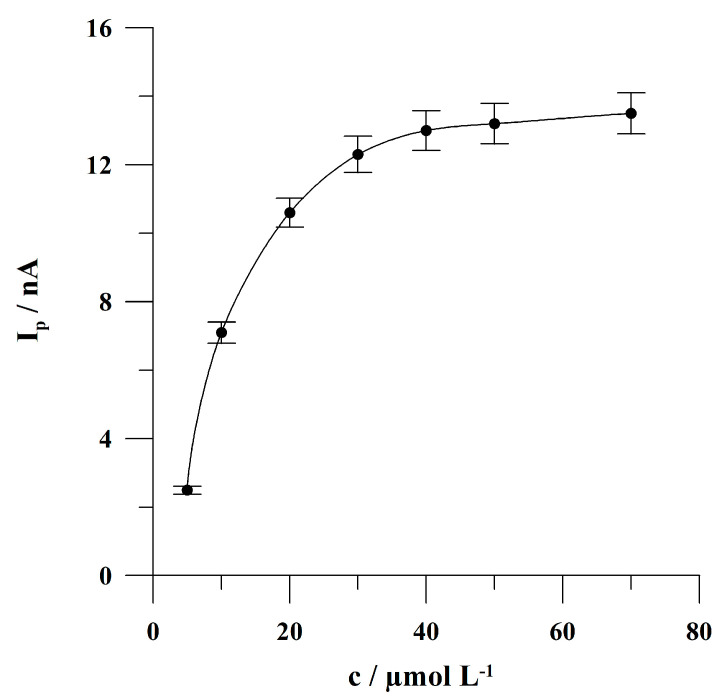
Effect of cupferron concentration on U(VI) signal. U(VI) concentration: 5 × 10^−8^ mol L^−1^. Accumulation conditions: −0.7 V, 120 s. The error bars refer to the standard deviation (n = 3).

Cupferron and chloranilic acid have often been used as complexing agents in the course of uranium ion determinations by means of adsorptive stripping voltammetry [60]. Due to the fact that a higher and better-shaped analytical signal of uranium was obtained in the presence of cupferron, this complexing agent was selected for further research. The effect of cupferron concentration on the height of the U(VI) signal was investigated from 5 × 10^−6^ to 7 × 10^−5^ mol L^−1^. U(VI) ion concentration was 5 × 10^−8^ mol L^−1^. Accumulation conditions were −0.7 V, 120 s. The obtained results are shown in Figure 4. It was observed that U(VI) signal increased with cupferron concentration to 4 × 10^−5^ mol L^−1^ and then reached almost constant value at higher cupferron concentrations. The obtained results allow to conclude that U(VI) ions present in a sample solution and available for complex formation are almost completely complexed at the cupferron concentration of 4 × 10^−5^ mol L^−1^. Based on the presented results, further studies were performed at cupferron concentration equal to 4 × 10^−5^ mol L^−1^.

### 3.4. Activation Step’s Conditions

As was reported previously [50,51], activation of a surface of solid metal microelectrodes is a crucial step for obtaining well-shaped and reproducible analytical signals. An activation step was provided before each particular measurement from the analyzed solution and from solutions with the added determined species. As was previously observed, the application of only an initial activation step conducted from an analyzed solution has not been enough for obtaining well-shaped and high analytical signals of determined species during subsequent measurements in the presence of an analyte. The best results in terms of the sensitivity, reproducibility and shape of an analytical signal were obtained when the activation step was applied before each particular measurement. This measurement step is performed in a short time at high negative potential values. During the activation step, the surface of a solid lead–tin microelectrode was cleaned due to the following processes: (i) reduction of oxides that may be formed at the surface of working metal microelectrode and (ii) desorption of contaminants potentially adsorbed during the accumulation step. Optimization of both mentioned parameters—potential and time of activation—requires that it be performed for each analytical procedure.

The potential of activation step of solid lead–tin microelectrode was changed in a range from −1.0 to −3.0 V, and its influence on the uranium(VI) analytical signal was investigated. The concentration of U(VI) was 5 × 10^−8^ mol L^−1^. Activation time was 4 s. Accumulation conditions were −0.7 V, 120 s. It was found that uranium peak height increased with an increase of the activation potential towards more negative values, reaching the highest value in the range from −2.5 to −3.0 V, as presented in Figure S2 (in Supplementary Material). An activation potential of −2.5 V was chosen for further research.

The time of activation step was changed from 1 to 5 s in order to choose the most optimum value for uranium(VI) determination. The concentration of U(VI) was 5 × 10^−8^ mol L^−1^. Activation potential was −2.5 V. Accumulation conditions were −0.7 V, 120 s. The obtained results of this experiment are shown in Figure S3 (in Supplementary Material). Time of activation practically did not influence the results of measurements; however, taking into account the fact that the highest peak current was observed for 3 s, this value was chosen for further investigation.

### 3.5. Accumulation Conditions

**Figure 5:**
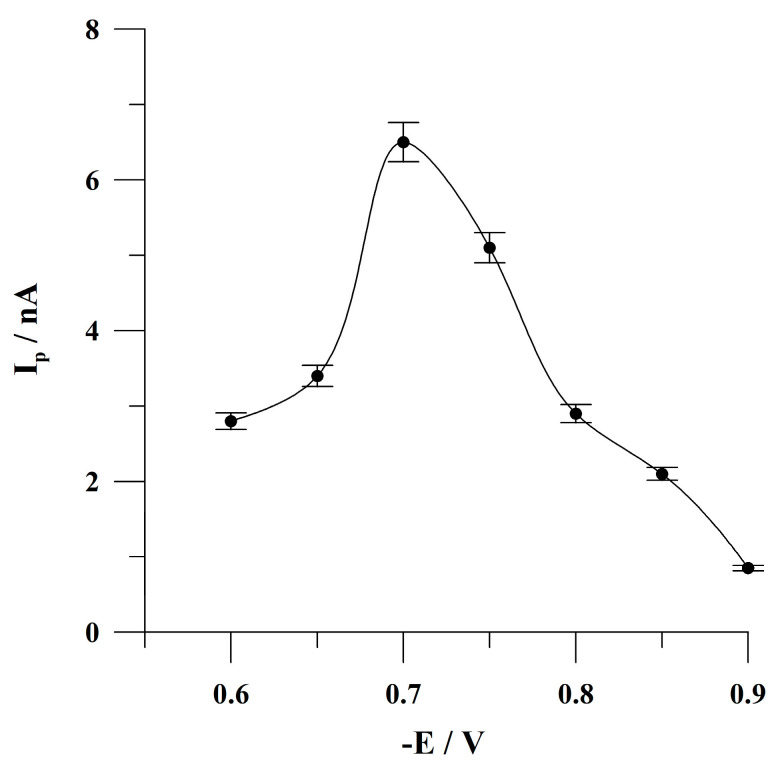
Effect of accumulation potential on U(VI) signal. U(VI) concentration: 3 × 10^−8^ mol L^−1^. Time of accumulation: 120 s. The error bars refer to the standard deviation (n = 3).

During the optimization of the analytical procedure, the influence of accumulation potential on uranium analytical signal was checked in order to select conditions at which the efficiency of an adsorption of U(VI)–cupferron complex on the surface of the lead–tin microelectrode was the highest. The accumulation potential effect on U(VI) peak current was checked from −0.6 to −0.9 V. U(VI) concentration and accumulation time were 3 × 10^−8^ mol L^−1^ and 120 s, respectively. The obtained results presented in Figure 5 show that the U(VI) signal increased from a potential of −0.6 V up to a potential of −0.7 V and then decreased at more negative potential values. The obtained results can be explained as follows: At accumulation potential within the range from −0.6 to −0.65 V, a slight oxidation of the microelectrode material may occur, as the value of −0.6 V is an anodic limit of the working range of the discussed lead–tin microelectrode. On the other hand, at more negative potential values, the adsorbed U(VI)–cupferron complex may undergo a partial reduction process. Taking the above into account, for further investigation the accumulation potential of −0.7 V was chosen.

**Figure 6:**
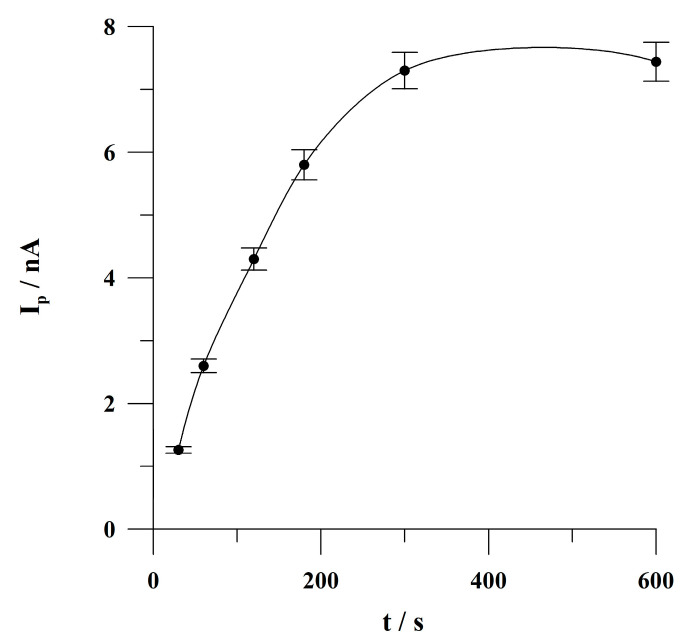
Effect of accumulation time on U(VI) peak current. U(VI) concentration: 2 × 10^−8^ mol L^−1^. Potential of accumulation: −0.7 V. The error bars refer to the standard deviation (n = 3).

The effect of accumulation time on uranium peak current was studied from 30 to 600 s. The obtained results are shown in Figure 6. U(VI) concentration was 2 × 10^−8^ mol L^−1^. It was observed that the U(VI) signal increased to 300 s and then remained constant at higher values of accumulation time. The increase of uranium analytical signal with accumulation time to 300 s is associated with the ability of adsorption of U(VI)–cupferron complex on the working microelectrode surface. The observed lack of increase of uranium analytical signal within the range from 300 to 600 s may indicate on the complete saturation of the microelectrode surface with U(VI)–cupferron complex. Further research was conducted at an accumulation time of 120 s to shorten the time for determinations; however, for the lowest U(VI) concentrations, accumulation time should be prolonged.

### 3.6. Frequency Optimization

**Figure 7:**
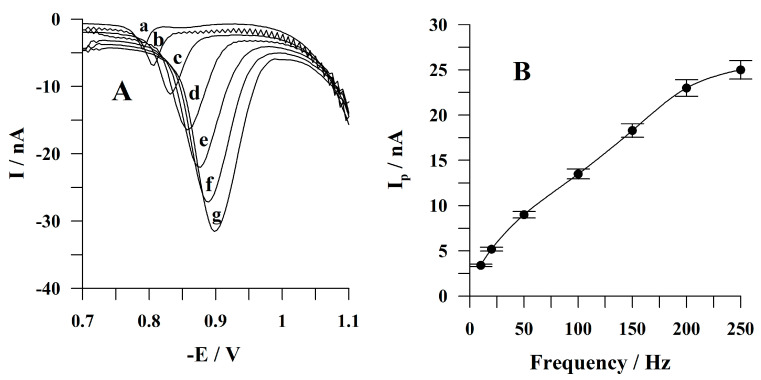
(**A**) The square wave uranium voltammograms obtained at various frequency values: (a) 10; (b) 20; (c) 50; (d) 100; (e) 150; (f) 200; (g) 250 Hz; (**B**) A dependence of U(VI) peak current on the frequency. U(VI) concentration: 1 × 10^−7^ mol L^−1^. Accumulation conditions: −0.7 V, 120 s. The error bars refer to the standard deviation (n = 3).

In order to determine U(VI) ions under the most favorable conditions, frequency as a main parameter of square wave voltammetry was optimized. Values of frequency were changed in the range from 10 to 250 Hz. The concentration of U(VI) was 1 × 10^−7^ mol L^−1^. Activation and accumulation conditions were −2.5 V, 3 s and −0.7 V, 120 s, respectively. The obtained results presented in the form of dependence of peak height on frequency are shown in Figure 7B. Corresponding voltammograms obtained during this investigations are shown in Figure 7A. The obtained results showed that uranium peak current increased within the whole range of studied frequency values; however, its increase was more slow, in the range from 200 to 250 Hz. The obtained dependence indicates on a high rate of the reduction process of U(VI)–cupferron complex; therefore, the registration of analytical signals by means of square wave technique is possible at high frequency values. Taking into account this observation and the fact that the background current increase was observed with increasing frequency, the value of 200 Hz was chosen for further research.

### 3.7. Calibration Studies

**Figure 8:**
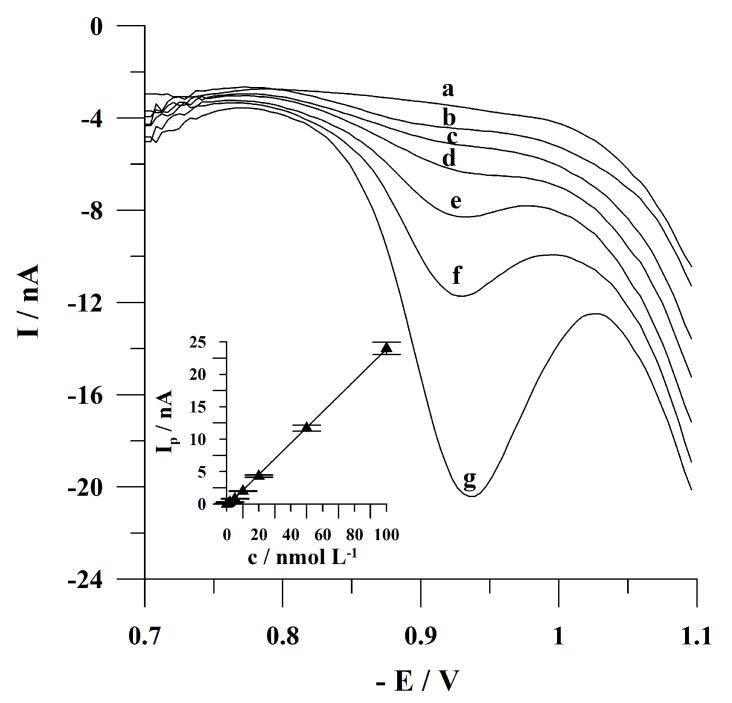
Square wave voltammograms registered for the lowest U(VI) concentrations: (a) blank; (b) 1 × 10^−9^; (c) 2 × 10^−9^; (d) 5 × 10^−9^; (e) 1 × 10^−8^; (f) 2 × 10^−8^; (g) 5 × 10^−8^ mol L^−1^. Accumulation conditions: −0.7 V, 120 s. Inset: linear calibration graph of U(VI) determination at 120 s of accumulation. The error bars refer to the standard deviation (n = 3).

The calibration plot for U(VI) determination at the proposed solid lead–tin microelectrode following 120 s of accumulation was a straight line from 1 × 10^−9^ to 1 × 10^−7^ mol L^−1^ and is described by the equation y = 0.2415x − 0.274; y and x are the peak current (nA) and U(VI) concentration (nmol L^−1^), respectively, with r (a linear correlation coefficient) equal to 0.999. Voltammograms obtained during calibration studies are shown in Figure 8. The linear calibration plot is shown in an insertion to Figure 8. The repeatability of the analytical signal of uranium determination at the solid lead–tin microelectrode was tested for two varied U(VI) concentrations. The signals obtained for seven successive measurements with the supporting electrolyte containing 2 × 10^−8^ or 5 × 10^−8^ mol L^−1^ of U(VI) were compared. The calculated values of RSD were equal to 3.5% and 3.3%, respectively. The detection limit (LOD) for U(VI) determination following an accumulation time of 120 s was equal to 3.9 × 10^−10^ mol L^−1^. LOD was calculated from the following expression: 3 s m^−1^, where s is the standard deviation for a low U(VI) concentration and m is a slope of the calibration plot. The obtained LOD is a lower value in comparison to the detection limit obtained using a solid lead microelectrode proposed previously [50]. The U(VI) quantification limit (LOQ) following an accumulation time of 120 s was calculated to be 1.5 × 10^−9^ mol L^−1^. LOQ was calculated from the expression 10 s m^−1^. The analytical characteristic of the developed procedure is presented in Table 1. The comparison of the analytical parameters of procedures of uranium determination using various microelectrodes by means of stripping voltammetry is presented in Table 2.

### 3.8. Stability of the Sensor

The stability in time of the proposed sensorpresented as a reproducibility of the uranium analytical signal was investigated for the supporting electrolyte containing 5 × 10^−8^ mol L^−1^ U(VI) by a consecutive analysis of a sample five months after the procedure was developed (n = 7). The calculated RSD value was equal to 5.4%.

Furthermore, a reproducibility of uranium analytical signal was checked between results obtained for five solid lead–tin microelectrodes prepared in a way described in Section 2.3 above. The RSD value calculated from the results obtained for several microelectrodes was equal to 4.6%.

The obtained results indicate good reproducibility of fabricated sensors. The data described above indicate that the solid lead–tin microelectrode is a more stable sensor for U(VI) determination as compared to modified and screen printed electrodes [61,62].

### 3.9. Interferences

The tolerable limits of foreign ions potentially present in natural water samples were investigated for a solution containing 5 × 10^−8^ mol L^−1^ U(VI) ions. The studies were conducted at an accumulation time of 120 s. The impact of the foreign ions on the U(VI) peak height is shown in Table 3. The obtained results indicate that U(VI) peak current was not disturbed by a majority of added ions. The only significant effect was noticed in the presence of a hundredfold excess of Sn(IV) and a tenfold excess of Mo(VI); however, such concentrations of Sn(IV) and Mo(VI) are rarely observed in environmental water samples. Interferences related to the presence of surfactants were investigated on the example of Triton X-100. It was observed that the addition of 0.1 and 0.5 mg L^−1^ of nonionic Triton X-100 caused a decrease of U(VI) peak current to 50% and 13% of its original value, respectively. Moreover, the impact of the presence of EDTA as a competitive complexing agent was studied. It was found that in the presence of 2 × 10^−5^ and 5 × 10^−5^ mol L^−1^ of EDTA, a decrease of U(VI) peak current to 42% and 20% of its output value, respectively, was observed. The above results lead to the conclusion that natural water samples containing high concentrations of natural organic matter need to be mineralized before actual analysis.

### 3.10. Analytical Applications

**Figure 9:**
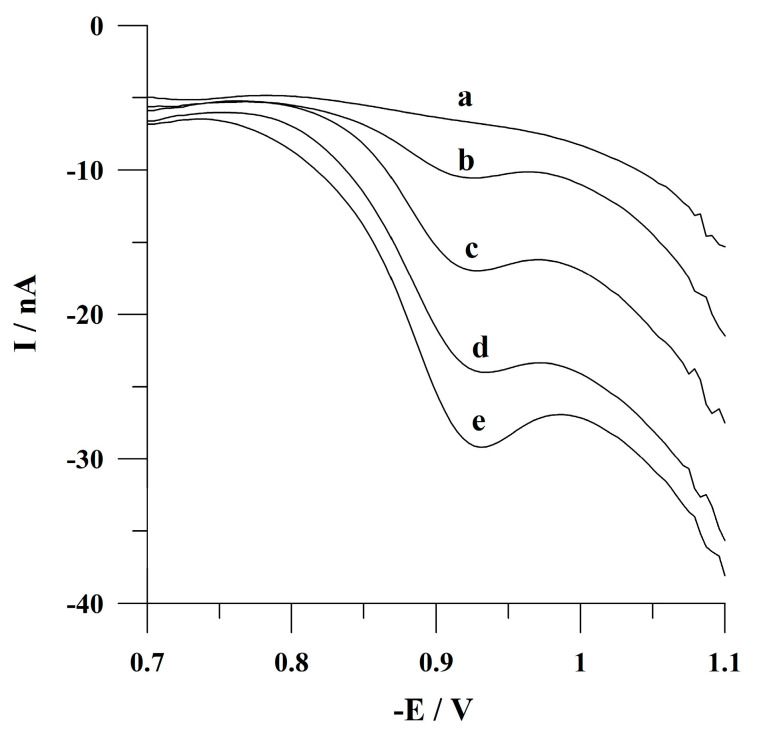
Square wave voltammograms obtained during analysis of certified reference material TM 25.5. (a) blank; (b) as (a) + 885 µL TM25.5 + 55.8 µL 1 mol L^−1^ NaOH; (c) as (b) + 1 × 10^−8^ mol L^−1^ U(VI); (d) as (c) + 1 × 10^−8^ mol L^−1^ U(VI); (e) as (d) + 1 × 10^−8^ mol L^−1^ U(VI). Accumulation time: 120 s.

The described AdSV procedure was applied for U(VI) determination in the natural certified Lake Ontario water reference material TM 25.5 (factor of dilution: 11). The determinations were performed using the standard additions method that inherently takes into account any matrix interferences that could affect the voltammetric response of determined species. The determination was repeated three times. The applied accumulation time was 120 s. After a mineralization described in Section 2.4 above, a sample of reference material was added to the electrochemical cell to make an elevenfold dilution. Additionally, 1 mol L^−1^ NaOH was used to adjust the pH of an analyzed solution. To determine uranium concentration in the analyzed material, three standard additions with a U(VI) concentration of 1 × 10^−8^ mol L^−1^ were used. Adsorptive stripping voltammograms obtained for U(VI) determination in a sample of certified lake water in reference material TM 25.5 are presented in Figure 9. The result of determination equal to 25.7 µg L^−1^ (1.08 × 10^−7^ mol L^−1^) (RSD 4.1 %; n = 5) is consistent with the certified value of 26.6 µg L^−1^ (±) 2.1 (1.12 × 10^−7^ mol L^−1^ ± 8.82 × 10^−9^). The obtained result confirms the possibility of using the developed procedure for analysis of the environmental water samples.

## 4. Conclusions

This paper demonstrates the application of a new voltammetric sensor, the solid lead–tin microelectrode. The presented results indicate that the proposed microelectrode can be successfully utilized for U(VI) determination by adsorptive stripping voltammetry. The developed procedure is characterized by two orders of magnitude linear dynamic range, low detection and quantification limits and a satisfactory precision and selectivity of U(VI) determination. The obtained detection limit was calculated to be 3.9 × 10^−10^ mol L^−1^ and is lower than a previously reported value for a solid lead microelectrode [50]. Lower values of detection limits of uranium determination using microelectrodes were reported by Khadro et al. and Korolczuk et al. in [48,49], respectively; however, in [48] a narrow range of a linearity of a calibration plot was obtained, while in a [49] uranium species were determined in double accumulation and double stripping step system. The described procedure can be used for analysis of natural water samples, which was proven by satisfactory results obtained during U(VI) determination in the certified lake water reference material.

The undoubted advantage of the described new solid lead–tin microelectrode is the possibility of its prolonged applicability that results from the way it is constructed. The step of metal film formation, crucial in the course of metal film electrodes, is omitted in the proposed procedure, which facilitates and shortens the measurement procedure and makes it more ecological. Another advantage is the possibility of carrying out the measurements without a solution deoxygenation. Thanks to the latter facts, the new voltammetric sensor, a solid lead–tin microelectrode, is an interesting and convenient alternative to using metal film electrodes. Furthermore, thanks to a greater reduction of the content of metallic lead as a toxic electrode material, the proposed solid lead–tin microelectrode is even more eco-friendly than a previously reported solid lead microelectrode [50]. The additional advantage of a solid lead–tin microelectrode is the fact that no surface modification is needed, and consequently, the developed procedure is relatively simple, fast and inexpensive.

## Figures and Tables

**Table 1:** Analytical features of the developed procedure.

Parameter	Value	Unit
Slope	0.2415	nA/nmol L^−1^
Intercept	0.274	nA
r	0.999	-
Linear range	1–100	nmol L^−1^
LOD	0.39	nmol L^−1^
LOQ	1.5	nmol L^−1^
RSD of analysis of 20 nmol L^−1^ U(VI) (n = 7)	3.5	%

LOD: detection limit; LOQ: quantification limit; RSD: relative standard deviation.

**Table 2:** Summary of analytical features of procedures of uranium determination using various microelectrodes by means of stripping voltammetry.

Working Electrode	Method	Linear Range [nmol L^−1^]	Detection Limit [nmol L^−1^]	Ref.
Au µE	ASV	100–10,000	1	[46]
Powder µE	CV	-	-	[47]
SPE µE	AdSV	0.021–0.042	0.0021	[48]
PbF µE	AdSV	0.1–5	0.031	[49]
Pb µE	AdSV	2–100	0.55	[50]
Pb-Sn µE	AdSV	1–100	0.39	[present paper]

Explanation of abbreviations: µE, microelectrode; ASV, anodic stripping voltammetry; CV, cyclic voltammetry; AdSV, adsorptive stripping voltammetry.

**Table 3:** Relative U(VI) signal after and before the addition of foreign ions. U(VI) concentration: 5 × 10^−8^ mol L^−1^. Accumulation time: 120 s.

Foreign Ions	Molar Excess of Foreign Ion	Relative Signal of U(VI)
Zn(II)	100	0.98
Mn(II)	100	1.02
Ni(II)	100	0.86
Cu(II)	100	0.90
Co(II)	100	0.63
Fe(III)	100	1.01
V(V)	100	0.91
Mo(VI)	5	0.81
	10	0.57
Sn(IV)	100	0.48

## Data Availability

No new data were created or analyzed in this study. Data sharing is not applicable to this paper.

## Supplementary Materials

The following supporting information can be downloaded at: https://www.mdpi.com/article/10.3390/s23052552/s1, Figure S1: SW voltammograms obtained for U(VI) determination at a concentration of 5 × 10^−8^ mol L^−1^ from: (a) a stirred solution; (b) an unstirred solution. Potential and time of accumulation: −0.7 V, 120 s. Figure S2: Effect of activation potential on U(VI) signal. U(VI) concentration: 5 × 10^−8^ mol L^−1^. Activation time: 4 s. Potential and time of accumulation: −0.7 V, 120 s. The error bars refer to the standard deviation (n = 3). Figure S3: Effect of activation time on U(VI) signal. U(VI) concentration: 5 × 10^−8^ mol L^−1^. Activation potential: −2.5 V. Potential and time of accumulation: −0.7 V, 120 s. The error bars refer to the standard deviation (n = 3).
