# Supplementary material for: A Novel Eco-Friendly and Highly Sensitive Solid Lead–Tin Microelectrode for Trace U(VI) Determination in Natural Water Samples
Source: Sensors (Basel). 2023 Feb 24;23(5):2552. doi: 10.3390/s23052552 (PMC10007126; doi:10.3390/s23052552)
Supplement: Supplementary file 1 [file sensors-23-02552-s001.zip › sensors-2201033-supplementary.pdf]

Supplementary Materials

A Novel Eco-Friendly and Highly Sensitive Solid Lead-tin Microelectrode for Trace U(VI) Determination in Natural Water samples

*Iwona Gęca[a], Mieczyslaw Korolczuk[a]

[1] *Institute of Chemical Sciences, Faculty of Chemistry, Maria Curie Sklodowska University, 20-031 Lublin, Poland*

* e-mail: iwona.geca@mail.umcs.pl

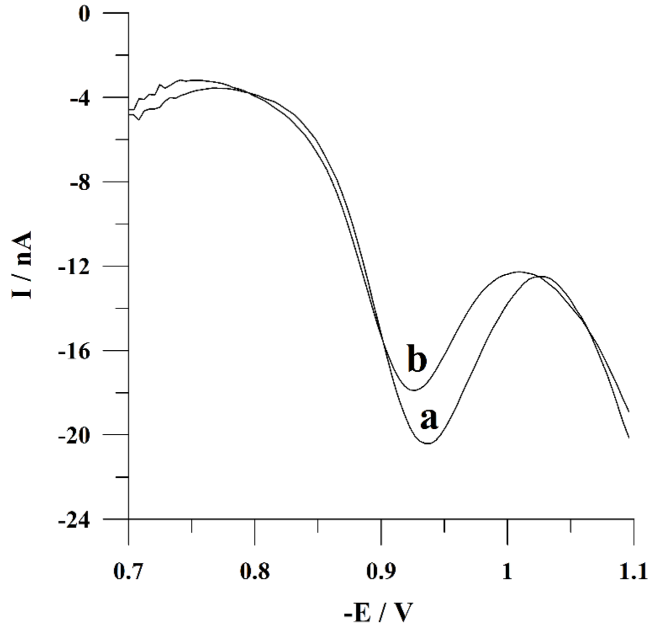

Figure S1. SW voltammograms obtained for U(VI) determination at a concentration of 5×10^{-8} mol L^{-1} from: a) a stirred solution; b) an unstirred solution. Potential and time of accumulation: -0.7 V, 120 s.

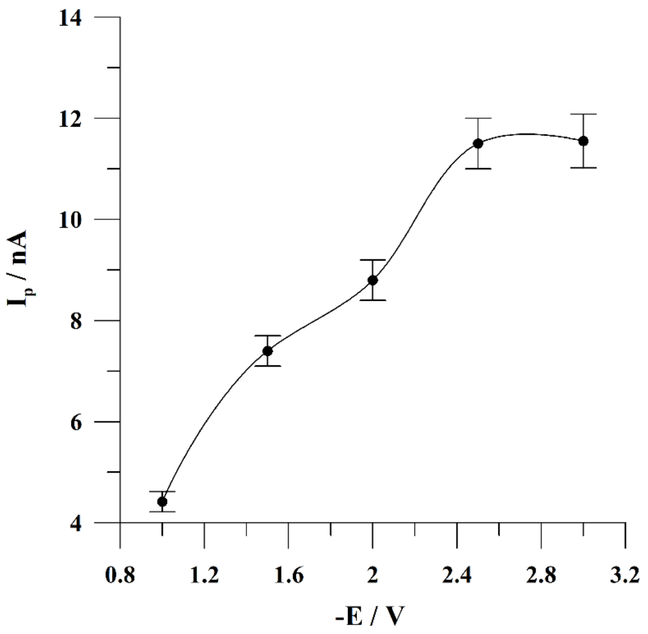

Figure S2. Effect of activation potential on U(VI) signal. U(VI) concentration: 5×10^{-8} mol L^{-1}. Activation time: 4 s. Potential and time of accumulation: -0.7 V, 120 s. The error bars refer to the standard deviation (n = 3).

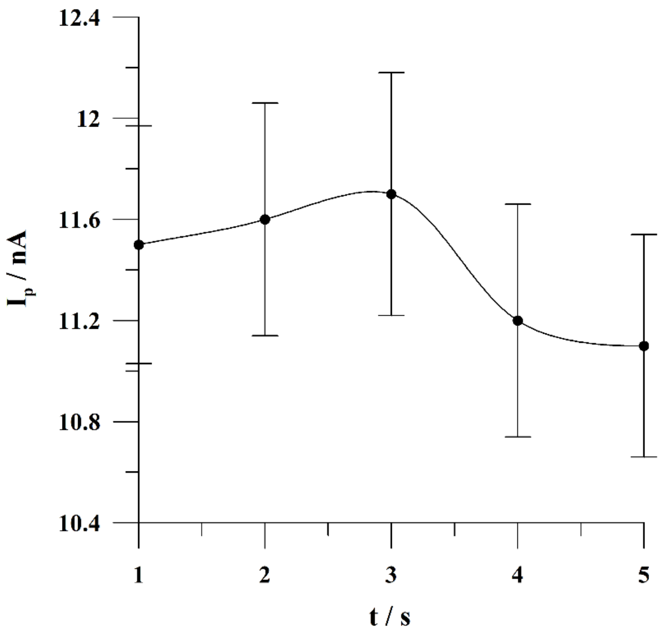

Figure S3. Effect of activation time on U(VI) signal. U(VI) concentration: 5×10^{-8} mol L^{-1}. Activation potential: -2.5 V. Potential and time of accumulation: -0.7 V, 120 s. The error bars refer to the standard deviation (n = 3).
